# Parity-Time Symmetry Breaking in Coupled Nanobeam Cavities

**DOI:** 10.1038/srep24487

**Published:** 2016-04-14

**Authors:** Senlin Zhang, Zhengdong Yong, Yuguang Zhang, Sailing He

**Affiliations:** 1 Centre for Optical and Electromagnetic Research, Zhejiang Provincial Key Laboratory for Sensing Technologies, JORCEP, East Building #5, Zijingang Campus, Zhejiang University, Hangzhou 310058, China; 2 Department of Electromagnetic Engineering, School of Electrical Engineering, Royal Institute of Technology (KTH), S-100 44 Stockholm, Sweden

## Abstract

The concept of parity-time symmetry (PT symmetry) originates from the canonical quantum mechanics and has become a hot topic recently. As a versatile platform to investigate the intriguing concept, both theoretical and experimental works in optics have been implemented. In this paper, the PT symmetry breaking phenomenon is investigated in a coupled nanobeam cavity system. An exceptional point is observed during the tuning of the gain/loss level and the coupling strength of the closely placed nanobeam pair. Unidirectional light propagation is investigated, as well as enhanced sensitivity of single particle detection in the vicinity of the exceptional point. The proposed system is easy to be integrated with photonic integrated circuits and can be strongly coupled to optical waveguides.

Since the notion that non-Hermitian Hamiltonians with PT symmetry can exhibit entirely real spectra was proposed by Bender *et al.* in 1998^1^, the PT symmetry concept has been extensively studied. In general, a Hamiltonian is PT symmetric provided that it commutes with the PT operator^1^, that is, its complex potential is subject to a spatial symmetry constraint 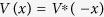. This complex potential can be easily implemented in the optical realm by modulating the real part and imaginary part of the refractive index to be an even function and odd function, respectively. Since the imaginary part of the refractive index represents gain or loss, it is easy to realize the above constraint through tuning the gain/loss level of the optical system.

One of the most fascinating features of a PT symmetric system is the existence of an exceptional point (EP), above which the PT symmetry is broken and the real spectra start to become complex. The appealing optical phenomena have resulted in much investigation and implementation in optics, both theoretically and experimentally. Basic theory and analytic results of PT symmetric structures are studied successively^2^,^3^. Optical systems including coupled optical waveguides^4^,^5^,^6^, photonic lattices^7^,^8^,^9^,^10^, plasmonics^11^, pumped lasers at EP^12^,^13^,^14^,^15^, nonlinear PT symmetric systems^16^,^17^, chaotic optical microcavity^18^, atom cavity composite system^19^, and whispering gallery modes^20^,^21^ have been proposed and explored. Applications such as laser absorbers^12^,^13^,^14^,^15^, single mode lasers^22^,^23^, unidirectional light propagation^24^,^25^,^26^, and sensitivity enhanced particle detectors^27^ have been validated.

Despite the extensive research, the PT symmetry phenomenon has not been discussed in nanobeam cavities, which have high quality factors (Q factors), small mode volume, compatibility with standard complementary metal-oxide semiconductor (CMOS) technology and ease of coupling with photonic integrated waveguides^28^.

In this paper, we propose a coupled nanobeam cavity system that shows a sharp symmetry transition. The system is based on a silicon-on-insulator (SOI) platform and nanobeam cavities are utilized. Compared to a previous work^21^, the present system has the advantage of e.g. easy coupling with photonic integrated circuits and simple fabrication process. The factors determining the PT symmetry transition are considered, and potential applications such as unidirectional light propagation and single particle sensing are also discussed.

## Results

### Structure of nanobeam cavity

**Figure 1:**
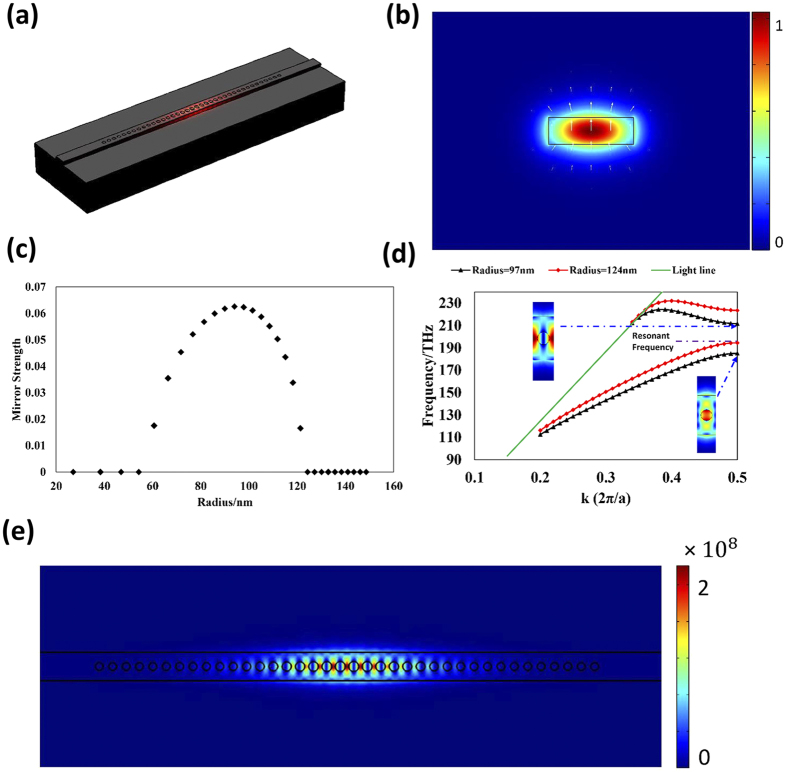
(**a**) Geometric structure of the PCNC. The upper cladding is omitted for illustration. (**b**) The distribution of the electric field of the 220 × 700 nm waveguide. (**c**) Mirror strength versus different radii of holes etched into the silicon core. (**d**) Bandgaps for radii of 97 nm and 124 nm of the unit cell. The resonant mode is around the lower band of the 124 nm-radius cell. The electric field distributions for the so-called dielectric mode and air mode are shown in the inset. (**e**) The light intensity of the resonant mode at 190.31 THz with a quality factor (Q factor) of about 64000.

The parameters of a one-dimensional photonic crystal nanobeam cavity (PCNC) are determined according to ref. 29. The thickness and width of the silicon core are 220 nm and 700 nm, respectively. The refractive index of the silicon core is 3.47 while that of the silica buffer layer and the polymer cladding are both 1.46. There are a total of 38 holes etched into the silicon layer with 10 holes whose dimensions linearly decrease from the center to both ends and 9 following holes with constant radii. The lattice constant of the PCNC is 330 nm. The geometry and vital characteristics of the PCNC are illustrated in Fig. 1(a). Figure 1(b) depicts the fundamental mode of the core waveguide (220 × 700 nm). High Q factor can be achieved if the electric field attenuation has a Gaussian shape from the center to the outermost part of the cavity along the horizontal direction, where the attenuation coefficient is defined as the so-called mirror strength^29^. In order to determine the size of the holes, series of band diagram simulations were performed using the Finite Element Method (FEM) to calculate the mirror strength of different radii. The analytic equation defining the mirror strength is 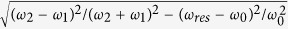, where ω_1_, ω_2_, ω_0_ and ω_*res*_ are the dielectric band edge, air band edge, middle frequency and resonant frequency^29^. The calculated mirror strength is shown in Fig. 1(c) as the hole radius varies, and the radii of the holes are chosen to be 124 nm in the center and 97 nm (with the largest reflecting capability) in the end according to Fig. 1(c). The band diagram for the central and outermost holes are shown in Fig. 1(d), and the optical intensity distribution of the dielectric band edge and air band edge are shown in the inset. Figure 1(e) characterizes the resonant mode at 190.31 THz, which displays a Q factor of about 64000. One thing to note is that the Q factor can be further elevated (by up to several millions) through increasing the number of holes. However, since the Q factor is not so crucial in the consideration of PT symmetry, only 38 holes in total are chosen for simplicity of numerical calculations.

### Coupled Nanobeam Cavity Pairs

**Figure 2:**
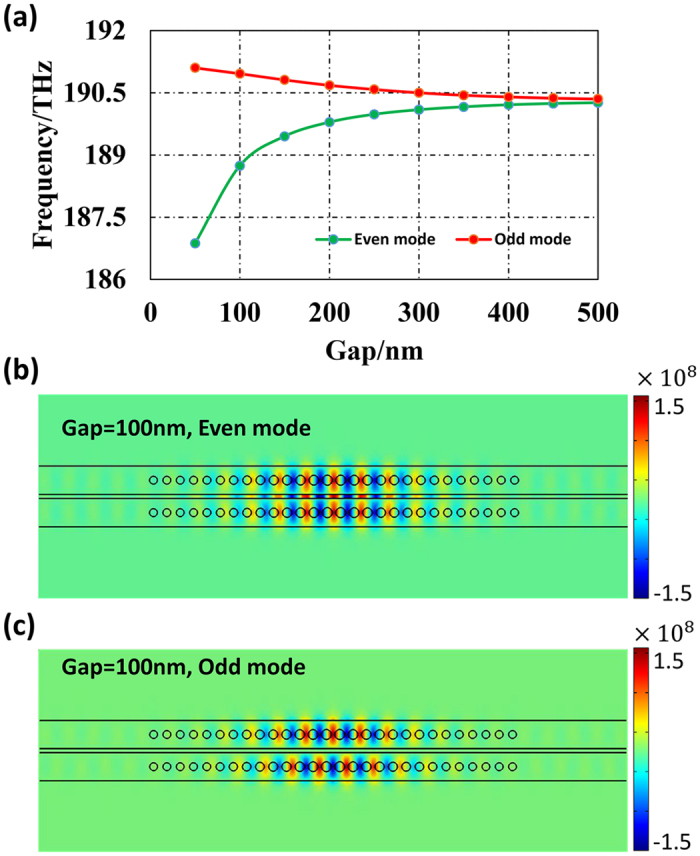
(**a**) The frequency detuning with variant gap between the two PCNCs without any gain/loss. (**b**,**c**) depict the electric field distributions of the even and odd modes, respectively.

Two closely placed PCNC pair without any gain/loss will show frequency splitting resulting from the coupling between the cavities. To calculate the frequency detuning of the system under different gaps, two identical PCNCs with the above-mentioned parameters are placed closely. The FEM-based eigenfrequency analysis method (COMSOL Multiphysics) is utilized and the calculated results are shown in Fig. 2. Figure 2(a) manifests the large frequency splitting of the PCNC pairs when placed closely. When the gap is 100 nm, the frequency detuning is as high as 2.22 THz. As the gap becomes larger, the coupling strength between the cavities declines and leads to smaller frequency detuning. Figure 2(b,c) characterize the two supermodes (the symmetric even mode and antisymmetric odd mode). As we can see from Fig. 2(a), the frequency of the even mode decreases quickly as the gap decreases, while the frequency change of the odd mode is quite mild. Such a phenomenon is due to the second-order cross- and self-coupling effects^31^.

### PT symmetry broken in PCNC pair

For weakly coupled cavities with modal gain/loss, the governing coupled mode equations for the two supermodes (which will be even or odd no longer) are as follows:^2^,^30^


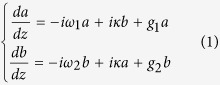


where a and b are the respective resonant modal amplitudes of the two cavities, ω_1_ and ω_2_ are the related eigenfrequencies, *κ* is the coupling strength between the two cavities and *g*_(1,2)_ is the gain/loss of the two nanobeam cavities (positive represents gain and negative for loss). The eigenfrequencies of the system can be derived as^23^


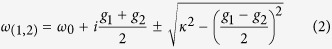


where ω_0_ is the eigenfrequency of the separated nanobeam cavities (assuming both nanobeam cavities are identical when no gain/loss). Apparently, the eigenfrequencies of the supermodes not only depend on the coupling strength, but also are affected by the gain/loss contrast, defined as 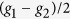, of the PCNC pair^23^.

**Figure 3:**
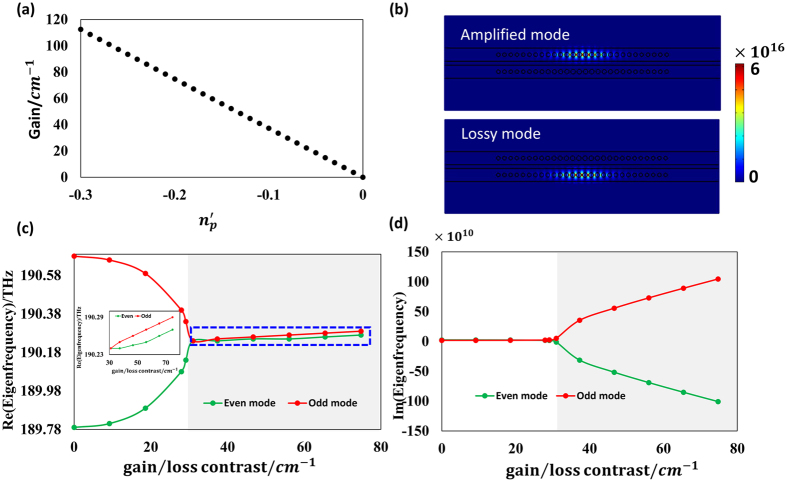
The calculated PT symmetry transition as the gain/loss contrast is finely tuned. The gain is implanted in the upper PCNC alone while the lower PCNC suffers loss. (**a**) The relation between the imaginary parts of the polymer embedded in the holes and the resulting gain. (**b**) The field distribution of the modes when the PT symmetry is broken with gain/loss contrast = 31 cm^−1^. The modes experiencing gain and loss are shown in the upper and lower parts of (**b**) separately. (**c**,**d**) depict respectively the evolution of the real and imaginary parts of the eigenfrequencies of the PCNC pair when gain/loss contrast varies. (The gap between the PCNC pair is 200 nm). The PT symmetry is broken in the shaded regions. The inset of (**c**) shows the enlarged view with much smaller variation range along the vertical axis.

Theoretically, the EP is reached when the gain and loss of the coupled cavities system are balanced, and the gain/loss contrast is matched with the coupling strength between the cavities, that is 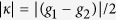. In order to study the PT symmetry broken in the PCNC pair, the relation between the eigenfrequency of the system and the gain/loss contrast and coupling strength is considered. The absolute value of the two artificial gain/loss values are set to be equal (i.e., 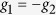) to ensure that the gain and loss of the system are balanced. Generally, such a gain or loss can be achieved through quantum well lasers^7^, photorefractive structures^7^ and erbium doping^21^,^26^ at the C band. The calculated PT symmetry transition under variable gain/loss contrast with specified coupling strength is shown in Fig. 3. The results shown below are obtained through the eigenfrequency analysis method of COMSOL Multiphysics. The gain or loss of the system is achieved by setting the imaginary part of the refractive index of the polymer (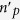) embedded in the holes to be negative or positive, respectively. Figure 3(a) shows the relation between the imaginary part of the polymer and the resulting gain of the system. Figure 3(c,d) illustrate the evolution of the eigenfrequencies of the two supermodes under variable gain/loss contrast when the gap between the two PCNCs is set to be 200 nm. Apparently, the EP is reached when the gain/loss contrast is about 31 cm^−1^. In such a case, both the real part and imaginary part of the eigenfrequencies coalesce. When the gain/loss contrast is over the transition threshold (the shaded region in Fig. 3(c,d)), the imaginary part of the eigenfrequencies bifurcate and one of the mode experiences gain (negative imaginary part of eigenfrequency) while the other experiences damping (positive imaginary part of eigenfrequency). Figure 3(b) shows the mode distribution when the PT symmetry is broken. One can see the distinct localization of the supermodes under this condition clearly (note that these supermodes are not even or odd any more due to the gain and loss introduced in the two PCNCs, but we will still call them “even/odd modes” for smooth transitions). This is quite abnormal compared with conventional coupled PCNC pairs. As the gain/loss contrast continues to increase, the “even mode” (amplified mode) experiences a larger amplification while the “odd mode” (lossy mode) keeps damping.

**Figure 4:**
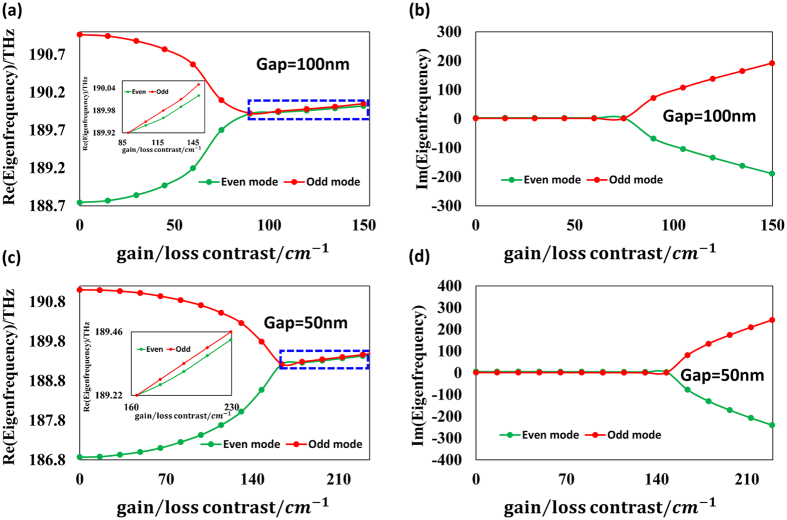
(**a**,**b**) illustrate the PT transition when the gain/loss contrast is finely tuned with the gap between the coupled cavities fixed to 100 nm, while (**c**,**d**) are the case when gap = 50 nm. The insets of (**a**,**c**) depict the enlarged view of the PT symmetry broken regions in (**a**) and (c) with much smaller variation range along the vertical axis.

In order to inspect the relation between the transition point and the coupling strength, the evolution of the eigenfrequencies is also calculated when the gap is 100 nm and 50 nm. Figure 4(a,b) show the transformation of the real and imaginary parts of the eigenfrequencies, respectively, when gap = 100 nm while (c) and (d) depict the case when gap = 50 nm. A larger transition threshold (gain/loss contrast) is observed as the gap declines.

**Figure 5:**
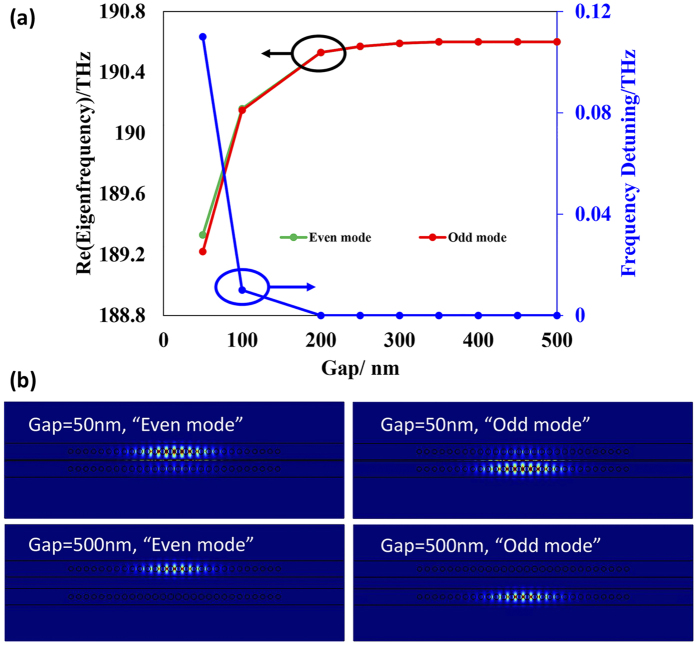
(**a**) The relation between the PT transition and coupling strength (smaller gap gives larger coupling strength) with balanced gain/loss. (**b**) Distribution of the electric field intensity under different gaps (50 nm and 500 nm).

The relation between the PT transition and coupling strength is further investigated with a specified gain/loss contrast. A gain/loss contrast of 31 cm^−1^ is chosen, as we have affirmed that the coupling strength matches this gain/loss contrast when the gap is 200 nm. Note that in this numerical example the PT symmetry remains when the gap is smaller than 200 nm and the PT symmetry is broken when the gap is larger than 200 nm. The variable coupling strength is represented by the different gaps between the two cavities and numerical calculation results are shown in Fig. 5. From Fig. 5(a), one can see that the exceptional point deviates when the coupling strength is larger than the gain/loss contrast (gap < 200 nm). Figure 5(b) shows that a portion of the amplified (even) mode’s energy is distributed in the “lossy (odd) cavity” and vice versa when the gap is 50 nm (due to the strong coupling). On the contrary, when the coupling strength is much smaller than the gain/loss contrast, both the amplified mode and lossy mode are well localized in each cavity as shown in the lower two figures of Fig. 5(b) (gap = 500 nm). The numerical calculations are well in accordance with the theoretical predications^32^. When the gap is small (50 nm), the coupling strength is larger than the gain/loss contrast and the electric field is not perfectly localized. When the gap is 500 nm, the gain/loss contrast is larger than the coupling strength and the PT symmetry is broken. Consequently the electric field is well confined in the gain or loss cavity for the “even mode” or “odd mode”. This phenomenon can be understood as follows. When the PT symmetry is unbroken (gap = 50 nm), the energy of the gain cavity can flow into the loss cavity rapidly to compensate the loss and this leads to the overlap of the two supermodes. In the broken case (e.g. gap = 500 nm), the coupling between these two cavities is weak and the loss of the lower cavity cannot be compensated by the flow of the energy from the gain cavity, which will result in two localized fields (one with gain and the other with loss)^21^.

### Unidirectional Light Propagation

Unidirectional light propagation, has been studied extensively^33^,^34^,^35^,^36^,^37^ since it is one of the key components for realizing on-chip optical signal processing. The beneficial localization characteristic of the PT symmetric nanobeam cavities makes this a promising concept for achieving unidirectional light propagation (through breaking the PT symmetry) with the help of gain saturated nonlinearity^26^.

**Figure 6:**
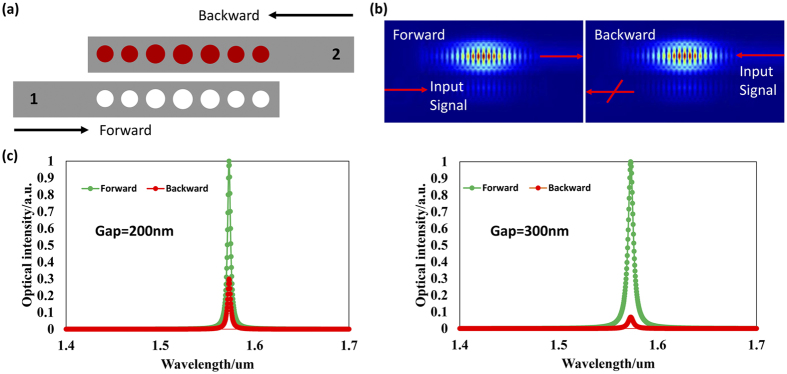
(**a**) The configuration for unidirectional light propagation. The PT symmetry is broken with gap = 200 nm. The other parameters for the gain/loss PCNCs are the same as those for Fig. 1(e). The upper nanobeam cavity with red holes is the gain cavity, and the lower one is the loss cavity. The direction (1 → 2) is defined as the forward direction and the (2 → 1) direction is backward. (**b**) The electric mode distribution under the forward and backward light propagation. (**c**) The transmission spectrum when the gap between the two coupled PCNCs is 200 nm or 300 nm. The gain/loss contrast is about 37 cm^−1^.

The numerical results using Finite Difference Time Domain (FDTD) are shown in Fig. 6. Figure 6(a) depicts the configuration of the system used in the calculation. The direction that light is injected from the left port (port 1) of the loss cavity is defined as the forward direction (1 → 2) while the direction that light is injected from the right port (port 2) of the gain cavity is the backward direction (2 → 1). The two eigenfrequencies (fundamental modes) of the cavities used here with no gain are around 1570 nm. When the system is in broken symmetry, the two eigenfrequencies coalesce as discussed before, and the transmission spectrum in the forward/backward direction is shown in Fig. 6(c). Figure 6(b) illustrate the unidirectional light propagation feature when the system is in a broken-symmetry status. The light is well confined in the gain cavity no matter which port (1 or 2) the light is injected into. Here we note that the gain is easier to saturate in the backward case due to the larger optical power since the light is directly injected into the gain cavity compared with the forward case (in which only a portion of light is coupled to the gain cavity). This will lead to a lower gain level in the backward case compared with the forward case^26^. Thus, provided that the system works in the gain-saturated nonlinear region, the light that is input from port 2 (backward) will possess a much weaker transmission to output port 1 (the red curve) than that of the forward case (the green curve) as shown in Fig. 6(c). We also note that such a gain-saturation-induced unidirectional light propagation in fact cannot function as a real isolator because such a “nonlinear isolator” cannot block a certain set of backward light when a forward signal is present^36^,^37^. However, as previous works^26^ done, the “isolation ratio”, defined as the ratio of the detected powers of the forward to backward transmissions, is calculated and is affected by the coupling strength (gap between the cavities) as shown in Fig. 6(c). The isolation ratio of the system is 5.24 dB and 11.63 dB for gap = 200 nm (strong coupling strength) and 300 nm (weak coupling strength), respectively (the PT symmetry is broken in both cases). This can be intuitively understood as follows: In the strong coupling case, larger portion of the backward optical signal can be coupled to the loss cavity than the weak coupling case. The calculated results matches well with the experimental results in ref. 26 but with a higher “isolation ratio”. Otherwise, the realization of unidirectional light propagation here outperforms previous works^21^,^26^ in terms of its compact feature size and excellent coupling capability with photonic integrated circuits.

### Enhanced sensitivity for single particle induced perturbation detection

An entirely passive nanobeam cavity has been implemented to sense the perturbation of the environment around the cavity based on the detection of the frequency shift. However, the sensitivity of such a sensor is proportional to the disturbance (ε ~ the effective refractive index perturbation in most cases). We will show that by operating around the EP the sensitivity can be enhanced massively (sensitivity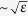) due to the sharp transition in the vicinity of the EP^27^. Therefore, a sensor based on the PT symmetry broken concept can be implemented to detect a tiny perturbation induced by a single particle and show better performance than a conventional sensor.

**Figure 7:**
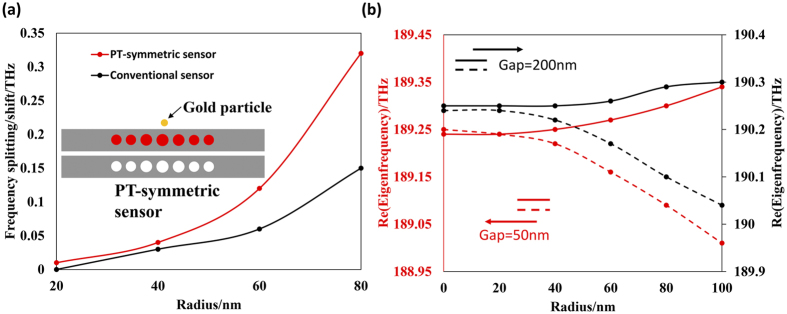
(**a**) The drastic frequency splitting of sensors against single particle perturbation in PT symmetric configuration and relatively gentle variation of frequency in single PCNC. The gold particle is placed in the vicinity of the gain cavity as shown in the inset. (**b**) The frequency splittings under different coupling strengths (i.e., different gaps) in PT symmetric sensors.

In order to examine the functionality of the PT-symmetric sensor, the coupled PCNC pair with a gap of 50 nm working in the vicinity of EP (the gain/loss contrast is about 166 cm^−1^) are utilized. Since the disturbance has a complex value in practical situations^27^,^38^, we use a single gold particle as the target for sensing. A larger gold particle will result in larger disturbance (larger loss which will break the balance between the coupling strength and gain/loss contrast more seriously) and lead to a larger frequency splitting. A gold particle is placed in the vicinity of the active PCNC with a gap of 10 nm. The FEM is used to find the eigenfrequencies of the system when the dimension of the gold particle varies. Figure 7(a) shows the performance comparison between a PT-symmetric configured sensor and a conventional sensor. For the conventional sensor, a gold particle is also placed adjacent to a single PCNC (without gain/loss) with a gap of 10 nm. The advantage of the PT-symmetric sensor over the conventional sensor can be clearly seen from the drastic change of frequencies due to the variation of the geometric size of the gold particle. The sensitivity of the PT-symmetric sensor is more than twice as large as that of the conventional sensor when the radius of the gold particle changes from 20 nm to 80 nm. Theoretically, the sensitivity not only depends on the disturbance, but also is affected by the gain/loss contrast (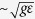) in the PT symmetric sensor^38^. Figure 7(b) shows the situation when the coupling strength of the PT symmetric sensor varies. The calculated results show that the sensor with a stronger coupling strength, i.e., the system needs a larger gain/loss contrast to realize the broken PT symmetry, possesses a higher sensitivity. In addition, the capability of enhancing the sensitivity of PCNCs applies to other judiciously designed ultrahigh-sensitive PCNC sensors provided that the PCNCs are tuned to work at the EP.

## Conclusion

The PT symmetry breaking phenomenon has been investigated in coupled nanobeam cavities. The dependence of the transition threshold on the gain/loss contrast and coupling strength has been considered and we have found that larger coupling strength will lead to larger transition threshold, which is well consistent with the theoretical predictions. The realization of unidirectional light propagation has been discussed, as well as the enhanced sensitivity for single particle detection. The compatibility of the nanobeam cavities with the CMOS technology and convenience of coupling with photonic integrated waveguides make the structure proposed here an excellent platform for on-chip optical signal processing and nanoparticle/biomolecule detection.

## Methods

The nanobeam cavities used in this paper is based on a SOI platform. The thickness of the silicon is 220 nm and the width of the silicon core is chosen to be 700 nm. Holes are filled with a polymer material, the real part of the refractive index of which is 1.46. The imaginary part of the material is set to be positive to represents loss and negative for gain. The refractive index of the upper-cladding material and the buffer layer are also 1.46 (the loss is neglected). 38 holes in total are chosen to form the PCNC. The eigenfrequencies of the single PCNC, coupled PCNC pair (with or without gain/loss) and the variation of the eigenfrequencies of the PT-symmetric sensors are investigated with the FEM-based eigenfrequency analysis method using COMSOL Multiphysics. All the boundaries are set as scattering boundary conditions. The unidirectional light propagation feature of the proposed system is studied using three-dimensional FDTD. The built-in Lorentz material model is used for the gain/loss materials. The Perfectly Matched Layers (PML) method is used for the boundary treatment of the calculation region.

## Additional Information

**How to cite this article**: Zhang, S. *et al.* Parity-Time Symmetry Breaking in Coupled Nanobeam Cavities. *Sci. Rep.* **6**, 24487; doi: 10.1038/srep24487 (2016).
